# Neuropeptide Trefoil Factor 3 Reverses Depressive-Like Behaviors by Activation of BDNF-ERK-CREB Signaling in Olfactory Bulbectomized Rats

**DOI:** 10.3390/ijms161226105

**Published:** 2015-11-30

**Authors:** Jiali Li, Yixiao Luo, Ruoxi Zhang, Haishui Shi, Weili Zhu, Jie Shi

**Affiliations:** 1 National Institute on Drug Dependence, Peking University, Beijing 100191, China; lijiali@bjmu.edu.cn (J.L.); luoyx@bjmu.edu.cn (Y.L.); zrx@bjmu.edu.cn (R.Z.); shhsh001@bjmu.edu.cn (H.S.); 2 Department of Pharmacology, School of Basic Medical Sciences, Peking University Health Science Center, Beijing 100191, China; 3 Beijing Key Laboratory on Drug Dependence Research, Beijing 100191, China; 4 The State Key Laboratory of Natural and Biomimetic Drugs, Beijing 100191, China; 5 Key Laboratory for Neuroscience of the Ministry of Education and Ministry of Public Healthy, Beijing 100191, China

**Keywords:** TFF3, olfactory bulbectomy, depression, BDNF, ERK, CREB

## Abstract

The trefoil factors (TFFs) are a family of three polypeptides, among which TFF1 and TFF3 are widely distributed in the central nervous system. Our previous study indicated that TFF3 was a potential rapid-onset antidepressant as it reversed the depressive-like behaviors induced by acute or chronic mild stress. In order to further identify the antidepressant-like effect of TFF3, we applied an olfactory bulbectomy (OB), a classic animal model of depression, in the present study. To elucidate the mechanism underlying the antidepressant-like activity of TFF3, we tested the role of brain-derived neurotrophic factor (BDNF)-extracellular signal-related kinase (ERK)-cyclic adenosine monophosphate response element binding protein (CREB) signaling in the hippocampus in the process. Chronic systemic administration of TFF3 (0.1 mg/kg, i.p.) for seven days not only produced a significant antidepressant-like efficacy in the OB paradigm, but also restored the expression of BDNF, pERK, and pCREB in the hippocampal CA3. Inhibition of BDNF or extracellular signal-related kinase (ERK) signaling in CA3 blocked the antidepressant-like activity of TFF3 in OB rats. Our findings further confirmed the therapeutic effect of TFF3 against depression and suggested that the normalization of the BDNF-ERK-CREB pathway was involved in the behavioral response of TFF3 for the treatment of depression.

## 1. Introduction

Neuropeptides participate in various behavioral and psychiatric processes, such as learning and memory, addiction, depressive, and anxiety disorders [1,2,3,4]. The trefoil factors (TFFs), a family of three small secretory polypeptides (TFF1, 2, and 3) in mammals and amphibians [5] are mainly synthesized and secreted by mucin-producing cells and play a critical role in mucosal defense and repair [6,7]. In addition to their prominent expression in epithelia of gastro-intestinal tissues, TFFs are also distributed in the central nervous system (CNS) [8]. TFF1 is broadly present in the hippocampus, cortex, and cerebellum [9]. There are nearly no data published concerning the TFF2 expression in brain, except that TFF2 was found in embryonic CNS [10]. TFF3 is mainly produced by the intestinal goblet cells and the antral mucous cells and is stable in the gastro-intestinal tract [7,11]. TFF3 is also detectable in bodily fluids, such as serum [12], urine [13], and cerebrospinal fluid [14]. In the CNS, TFF3 is initially found in the oxytocinergic neurons of the hypothalamus, and is then detected in other brain regions outside the hypothalamus including the hippocampus [15,16,17], which is important in regulating learning and memory as well as mood disorders [18,19]. The wide neural distribution of TFF3 suggests its potential roles in the CNS and related disorders.

Depression is a highly prevalent disorder that disturbs about 20% of the global population and produces a high rate of mortality [20]. Recently, we found that acute systemic TFF3 administration (0.1 mg/kg, i.p.) decreased the immobility time in both the tail suspension test and the forced swim test in mice, and reversed the depressive-like behaviors induced by chronic mild stress (CMS) in rats [21]. The olfactory bulbectomy (OB) rat model is a classic and widely used animal model of depression [22]. The removal of bilateral olfactory bulbs is known to cause a series of behavioral, physiological, and biochemical changes that are comparable to symptoms of depression in humans [23]. However, whether TFF3 could improve the abnormal behaviors induced by OB remains unknown.

An *in vitro* study has shown that the effects of TFF3 are mediated by the epidermal growth factor (EGF) receptor, which regulates the downstream pathways including the mitogen-activated protein kinase/extracellular signal-related kinase (MAPK/ERK) signaling [24]. Additionally, TFF3 could enhance the proliferation and migration of GES-1 gastric endothelial cells through the activation of ERK1/2 [25]. ERK is an important intracellular signaling pathway that is highly sensitive to stress and mood processing [26]. Brain-derived neurotrophic factor (BDNF), a peptide critical for axonal growth, neuronal survival, and synaptic plasticity, is an important regulator in the upstream pathway of ERK in depression [27]. A growing body of evidence from both postmortem and animal studies demonstrates that BDNF and its receptor, tropomyosin-related kinase B (TrkB), are involved in the pathophysiology and treatment of depression [27,28,29,30]. Additionally, ERK can regulate transcription by controlling the phosphorylation of the transcription factor cyclic adenosine monophosphate response element binding protein (CREB) [27]. CREB is involved in social isolation stress-induced emotional deficits [31]. In addition, chronic antidepressant treatment has also been shown to up-regulate the phosphorylation of CREB [28], and the increased CREB activation in rodent models of depression resulted in antidepressant-like effects [32]. This evidence suggested that the BDNF-ERK-CREB pathway might be involved in TFF3-mediated antidepressant-like effects.

In this study, we used OB rats to further confirm the antidepressant potential of TFF3 by the sucrose preference test (SPT), open field test (OFT) and forced swim test (FST). Furthermore, we assessed the alterations of the BDNF-ERK-CREB pathway in the hippocampus, aiming to explain the mechanism underlying the antidepressant-like effects of TFF3 in the rats OB paradigm.

## 2. Results and Discussion

### 2.1. Results

#### 2.1.1. Effects of Chronic TFF3 Administration on Olfactory Bulbectomy (OB)-Induced Depressive-Like Behaviors

**Figure 1:**
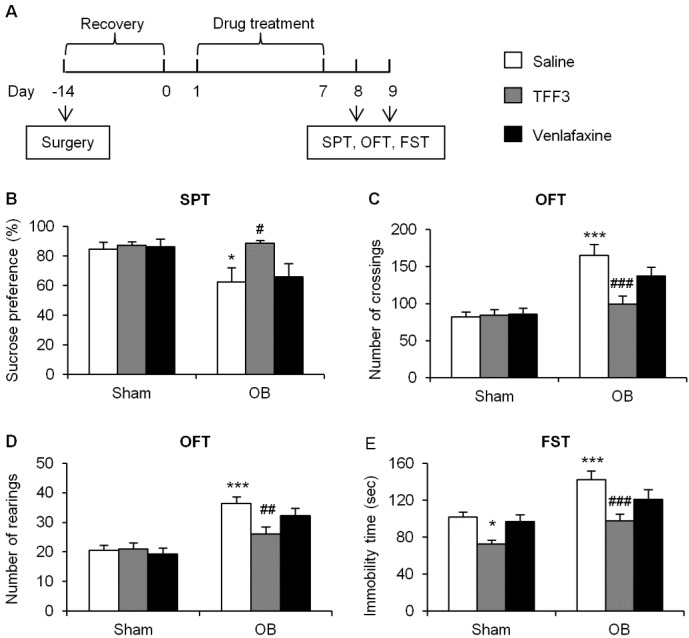
Chronic trefoil factor (TFF) 3 treatment produced antidepressant-like effects in olfactory bulbectomy (OB) rats. (**A**) Timeline of the experimental procedures. Decreased sucrose preference (**B**) in the sucrose preference test (SPT), increased crossings (**C**), and increased rearings (**D**) in an open field test (OFT), and increased immobility time (**E**) in the forced swim test (FST) were induced by the OB procedure but 7-day TFF3 treatment reversed the deficits. The data are expressed as mean ± SEM (*n* = 9 per group). * *p* < 0.05, *** *p* < 0.001, compared with sham-saline group; # *p* < 0.05, ## *p* < 0.01, ### *p* < 0.001, compared with OB-saline group.

As shown in Figure 1A, following a two-week recovery session after surgery, OB and sham rats were respectively treated with saline, TFF3 (0.1 mg/kg, i.p.), or venlafaxine (40 mg/kg, i.p.) once a day for seven days consecutively. The dose of TFF3 and venlafaxine was based on our previous experiments [21,33]. SPT, OFT and FST were then used to assess depressive-like behaviors or hyperactivity. All of the experimental processes were performed during the dark phase.

In SPT, with surgery (sham and OB) and treatment (saline, TFF3 and venlafaxine) as between-subject factors, the two-way analysis of variance (ANOVA) revealed the significant effects of surgery (*F*_(1,48)_ = 7.03, *p* < 0.05), but no significant effects of treatment (*F*_(2,48)_ = 2.91, *p* > 0.05) or surgery × treatment interaction (*F*_(2,48)_ = 2.17, *p* > 0.05). The *post hoc* analysis revealed that OB surgery significantly decreased the sucrose preference in rats with saline treatment (*p* < 0.05). After drug treatment for seven days, in OB rats TFF3 significantly increased the sucrose preference (*p* < 0.05, Figure 1B), while the classic antidepressant venlafaxine had little effect. In sham rats, TFF3 and venlafaxine had no effect on sucrose preference.

In OFT, the two-way ANOVA of the number of crossings showed significant effects of surgery (*F*_(1,48)_ = 34.08, *p* < 0.001) and treatment (*F*_(2,48)_ = 4.57, *p* < 0.05), as well as surgery × treatment interaction (*F*_(2,48)_ = 5.32, *p* < 0.01). The *post hoc* analysis revealed that the OB operation increased the number of crossings compared with the saline treated sham groups (*p* < 0.001). TFF3 administration for seven days reduced the number of crossings (*p* < 0.001). Venlafaxine tended to decrease the number of crossings but there was no significant effect (*p* > 0.05, Figure 1C).

Likewise, the two-way ANOVA of the number of rearings revealed the main effects of surgery (*F*_(1,48)_ = 38.42, *p* < 0.001) and treatment (*F*_(2,48)_ = 3.20, *p* < 0.05) as well as surgery × treatment interaction (*F*_(2,48)_ = 4.07, *p* < 0.05). The *post hoc* analysis revealed that the OB process increased the number of rearings (*p* < 0.001), while TFF3 administration decreased it (*p* < 0.01, Figure 1D). However, venlafaxine did not have a similar effect. Drug treatment on sham groups resulted no significant difference.

The FST was conducted the day after the OFT. The two-way ANOVA revealed significant effects of surgery (*F*_(1,48)_ = 23.65, *p* < 0.001) and treatment (*F*_(2,48)_ = 11.96, *p* < 0.001), but no significant surgery × treatment interaction (*F*_(2,48)_ = 0.74, *p* > 0.05). In sham rats, chronic TFF3 significantly reduced the immobility time compared with the saline-treated group (*p* < 0.05), whereas no significant change was observed in venlafaxine-treated rats. Compared with the sham-saline group, the OB procedure caused a significant increase in the immobility (*p* < 0.001). In OB rats, TFF3 reversed the immobility after one week of treatment (*p* < 0.001, Figure 1E).

The data from behavioral tests demonstrated that TFF3 produced therapeutic effects on hyperactivity and depressive-like behaviors induced by OB.

#### 2.1.2. Effects of Chronic TFF3 Administration on BDNF, Phosphorylation of ERK1/2 and CREB in the Hippocampus in the OB Rats

**Figure 2:**
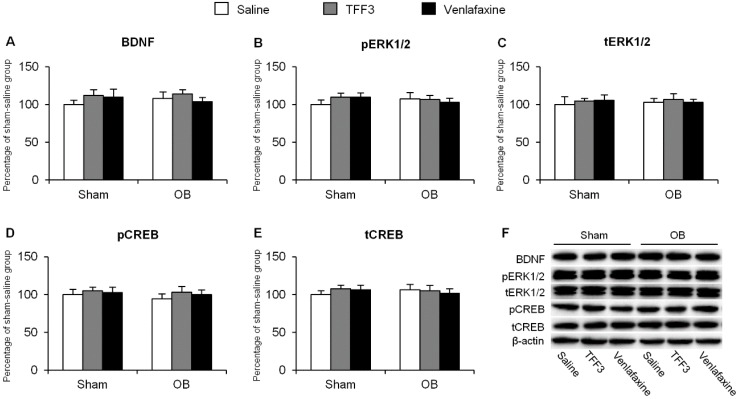
Effects of 7-day TFF3 treatment on Brain-derived neurotrophic factor (BDNF) expression and extracellular signal-related kinase (ERK) 1/2 and cyclic adenosine monophosphate response element binding protein (CREB) phosphorylation in hippocampal CA1. The levels of BDNF (**A**); pERK1/2 (**B**); tERK1/2 (**C**); pCREB (**D**) and tCREB (**E**) in CA1 were detected using Western blot; (**F**) Representative band intensities of the Western blot. The data are expressed as mean ± SEM (*n* = 6 per group).

Rats were decapitated 4 h after FST and brain tissues were collected. We determine the alterations of the BDNF-ERK-CREB pathway by Western blot. In hippocampal CA1, we found no significant change in the protein levels (Figure 2A–F).

**Figure 3:**
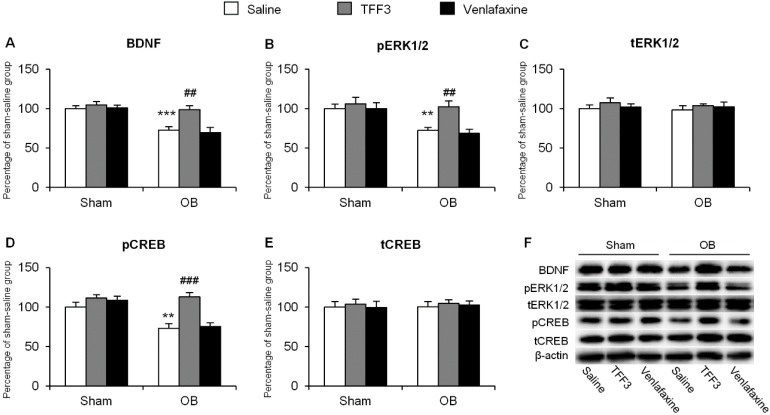
Effects of 7-day TFF3 treatment on BDNF expression and ERK1/2 and CREB phosphorylation in hippocampal CA3. The levels of BDNF (**A**); pERK1/2 (**B**); tERK1/2 (**C**); pCREB (**D**) and tCREB (**E**) in CA3 were detected using Western blot; (**F**) Representative band intensities of the Western blot. The data are expressed as mean ± SEM (*n* = 6 per group). ** *p* < 0.01, *** *p* < 0.001, compared with sham-saline group; ## *p* < 0.01, ### *p* < 0.001, compared with OB-saline group.

In CA3, the two-way ANOVA of BDNF data revealed significant effects of surgery (*F*_(1,30)_ = 30.09, *p* < 0.001) and treatment (*F*_(2,30)_ = 7.46, *p* < 0.01) as well as surgery × treatment interaction (*F*_(2,30)_ = 3.98, *p* < 0.05, Figure 3A). The two-way ANOVA of pERK1/2 data revealed significant effects of surgery (*F*_(1,30)_ = 15.33, *p* < 0.001) and treatment (*F*_(2,30)_ = 5.80, *p* < 0.01) but no significant effect of surgery × treatment interaction (*F*_(2,30)_ = 2.65, *p* > 0.05, Figure 3B). The two-way ANOVA of pCREB data revealed the significant effects of surgery (*F*_(1,30)_ = 18.61, *p* < 0.001) and treatment (*F*_(2,30)_ = 11.69, *p* < 0.001) as well as surgery × treatment interaction (*F*_(2,30)_ = 5.60, *p* < 0.01, Figure 3D). The *post hoc* analysis showed that OB decreased the levels of BDNF (*p* < 0.001, Figure 3A), pERK1/2 (*p* < 0.01, Figure 3B) and pCREB (*p* < 0.01, Figure 3D), which was reversed by seven-day consecutive TFF3 administration (*p* < 0.01 for BDNF and pERK1/2, Figure 3A,B; *p* < 0.001 for pCREB, Figure 3D). However, venlafaxine did not have such an effect. There was no significant change in the levels of total ERK1/2 and total CREB (Figure 3C,E).

**Figure 4:**
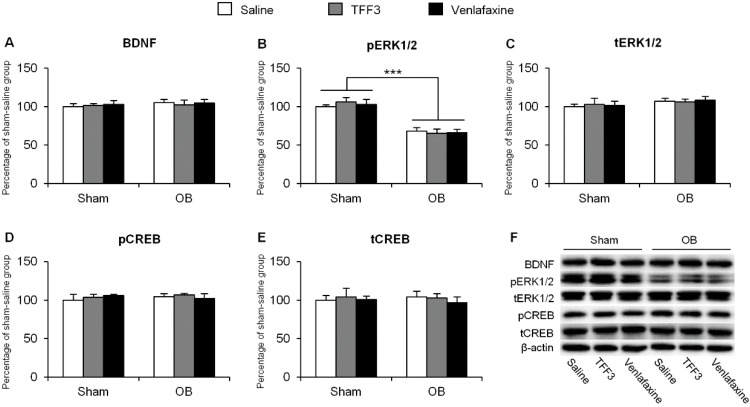
Effects of 7-day TFF3 treatment on BDNF expression and ERK1/2 and CREB phosphorylation in hippocampal DG. The levels of BDNF (**A**); pERK1/2 (**B**); tERK1/2 (**C**); pCREB (**D**) and tCREB (**E**) in DG were detected using Western blot; (**F**) Representative band intensities of the Western blot. The data are expressed as mean ± SEM (*n* = 6 per group). *** *p* < 0.001, compared with sham group.

In dentate gyrus (DG), the two-way ANOVA of pERK1/2 data revealed the significant effects of surgery (*F*_(1,30)_ = 79.03, *p* < 0.001) but no significant effects of treatment (*F*_(2,30)_ = 0.06, *p* > 0.05) or surgery × treatment interaction (*F*_(2,30)_ = 0.37, *p* > 0.05, Figure 4B). The level of pERK1/2 was decreased by OB operation (*p* < 0.001, Figure 4B) but 7-day TFF3 treatment had no significant effect. Besides, there was no significant change in the levels of BDNF, total ERK1/2, pCREB and total CREB (Figure 4A,C–E).

These results indicated that chronic TFF3 treatment reversed the decrease in the expression of BDNF, phosphorylation of ERK1/2, and CREB in the CA3 of OB rats. Considering the important roles of the BDNF/ERK/CREB pathway in depression, the therapeutic effects of TFF3 for depression in the OB paradigm may contribute to the renewal of the pathway.

#### 2.1.3. Effects of Tropomyosin-Related Kinase Receptor B (TrkB) Antagonist on Depressive-Like Behaviors in TFF3-Treated OB Rats

**Figure 5:**
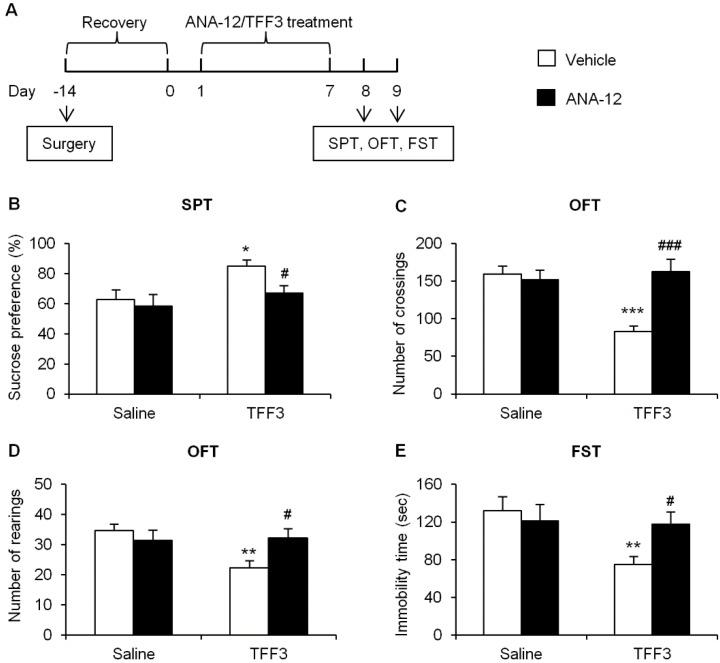
Blockade of tropomyosin-related kinase B (TrkB) by microinjection of ANA-12 into CA3 prevented the antidepressant-like effects of TFF3 in OB rats. (**A**) Timeline of the experimental procedures. Sucrose preference (**B**) in the sucrose preference test (SPT), crossings (**C**) and rearings (**D**) in the open field test (OFT), and immobility time in the forced swim test (FST) (**E**), were measured after consecutive drug treatment for 7 days. ANA-12 blocked the antidepressant-like effect of TFF3. The data are expressed as mean ± SEM (*n* = 7 per group). * *p* < 0.05, ** *p* < 0.01, *** *p* < 0.001, compared with saline-vehicle group; # *p* < 0.05, ### *p* < 0.001, compared with TFF3-vehicle group.

As TFF3 reversed the OB-induced down-regulation of BDNF signaling in hippocampal CA3. We used a 2 (saline or TFF3) × 2 (vehicle or ANA-12) factorial design to determine whether BDNF is involved in the antidepressant-like role of TFF3. As shown in Figure 5A, rats underwent surgeries of both OB and cannula implantation into CA3. Then rats were given a recovery period for 14 days. After recovery, OB rats were treated with TFF3 or saline for 7 consecutive days respectively. We blocked the BDNF receptor using the TrkB antagonist ANA-12 (1 μg) [34,35], and infusion into CA3 1 h before the saline or TFF3 treatment each day. After drug treatment, SPT, OFT, and FST were conducted.

In SPT, with TFF3 (saline and TFF3) and ANA-12 (vehicle and ANA-12) as between-subjects factors, the two-way ANOVA revealed significant effect of TFF3 (*F*_(1,24)_ = 6.39, *p* < 0.05), but no significant effects of ANA-12 (*F*_(1,24)_ = 3.31, *p* > 0.05) or TFF3 × ANA-12 interaction (*F*_(1,24)_ = 1.27, *p* > 0.05, Figure 5B). In OFT, the two-way ANOVA of the number of crossings revealed significant effects of TFF3 (*F*_(1,24)_ = 7.07, *p* < 0.05) and ANA-12 (*F*_(1,24)_ = 8.40, *p* < 0.01) as well as TFF3 × ANA-12 interaction (*F*_(1,24)_ = 12.04, *p* < 0.01, Figure 5C). The two-way ANOVA of the number of rearings revealed no significant effects of TFF3 (*F*_(1,24)_ = 4.14, *p* > 0.05) or ANA-12 (*F*_(1,24)_ = 1.30, *p* > 0.05), but significant TFF3 × ANA-12 interaction (*F*_(1,24)_ = 5.21, *p* < 0.05, Figure 5D). In FST, the two-way ANOVA revealed significant effect of TFF3 (*F*_(1,24)_ = 4.71, *p* < 0.05), but no significant effects of ANA-12 (*F*_(1,24)_ = 1.33, *p* > 0.05) or TFF3 × ANA-12 interaction (*F*_(1,24)_ = 3.69, *p* > 0.05, Figure 5E) on immobility time. The *post hoc* analysis showed that in OB rats, consecutive 7-day TFF3 treatment significantly increased sucrose preference (*p* < 0.05, Figure 5B), and decreased the numbers of crossings (*p* < 0.001, Figure 5C) and rearings (*p* < 0.01, Figure 5D) in OFT, and decreased the immobility time in FST (*p* < 0.01, Figure 5E) while ANA-12 pretreatment blocked these effects (*p* < 0.05, Figure 5B,D,E; *p* < 0.001, Figure 5C). These results indicated that BDNF in CA3 was necessary for the antidepressant-like action of TFF3 in the OB paradigm.

#### 2.1.4. Effects of ERK Phosphorylation Inhibition on Depressive-Like Behaviors in TFF3-Treated OB Rats

**Figure 6:**
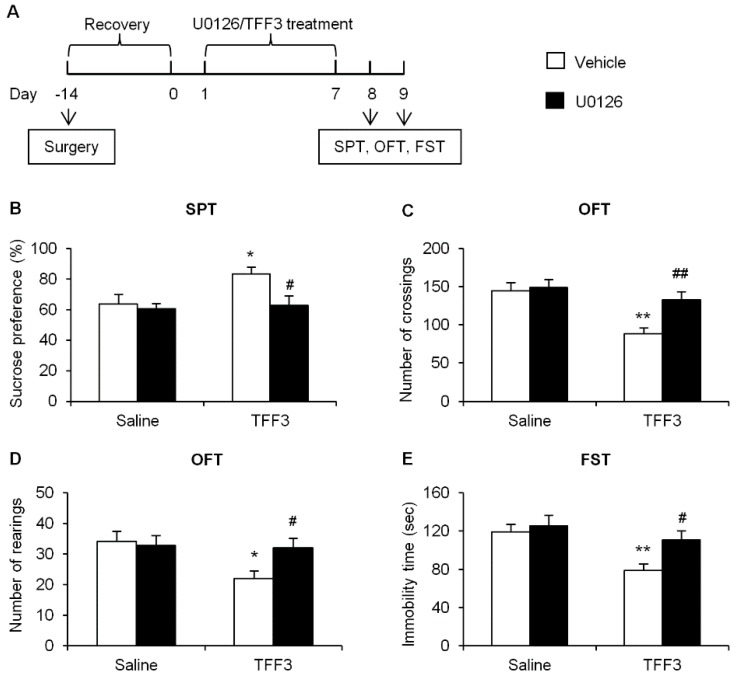
Inhibition of pERK in CA3 blocked the antidepressant-like effects of TFF3 in OB rats. (**A**) Timeline of the experimental procedures. Sucrose preference (**B**) in the sucrose preference test (SPT), crossings (**C**) and rearings (**D**) in the open field test (OFT), and immobility time in the forced swim test (FST) (**E**) were measured after 7-day drug treatment. U0126 blocked the antidepressant-like effects of TFF3 in OB rats. The data are expressed as mean ± SEM (*n* = 7–8 per group). * *p* < 0.05, ** *p* < 0.01, compared with saline-vehicle group; # *p* < 0.05, ## *p* < 0.01, compared with TFF3-vehicle group.

To further investigate the causal role of the ERK pathway in the antidepressant-like ability of TFF3 in OB rats, we used U0126, a specific inhibitor of ERK phosphorylation [36] in this study. The experimental procedure was shown in Figure 6A, in which U0126 (200 ng) was microinjected into CA3.

In SPT, with TFF3 (saline and TFF3) and U0126 (vehicle and U0126) as between-subjects factors, the two-way ANOVA revealed the significant effects of TFF3 (*F*_(1,27)_ = 4.28, *p* < 0.05) and U0126 (*F*_(1,27)_ = 4.81, *p* < 0.05), but no significant TFF3 × U0126 interaction (*F*_(1,27)_ = 2.64, *p* > 0.05, Figure 6B). In OFT, the two-way ANOVA of the number of crossings revealed significant effects of TFF3 (*F*_(1,27)_ = 13.10, *p* < 0.01) and U0126 (*F*_(1,27)_ = 5.87, *p* < 0.05), but no significant TFF3 × U0126 interaction (*F*_(1,27)_ = 3.86, *p* > 0.05, Figure 6C). The two-way ANOVA of the number of rearings revealed the significant effect of TFF3 (*F*_(1,27)_ = 4.40, *p* < 0.05), but no significant effects of U0126 (*F*_(1,27)_ = 1.98, *p* > 0.05) or TFF3 × U0126 interaction (*F*_(1,27)_ = 3.29, *p* > 0.05, Figure 6D). In FST, the two-way ANOVA of immobility time revealed significant effects of TFF3 (*F*_(1,27)_ = 9.11, *p* < 0.01) and U0126 (*F*_(1,27)_ = 4.57, *p* < 0.05), but no significant TFF3 × U0126 interaction (*F*_(1,27)_ = 1.96, *p* > 0.05, Figure 6E). The *post hoc* analysis showed that 7-day TFF3 treatment significantly increased sucrose preference (*p* < 0.05, Figure 6B), and decreased the numbers of crossings (*p* < 0.01, Figure 6C) and rearings (*p* < 0.05, Figure 6D) in OFT, and decreased the immobility time in FST (*p* < 0.01, Figure 6E), while U0126 blocked these effects (*p* < 0.05, Figure 6B,D,E; *p* < 0.01, Figure 6C). These results indicated that the ERK activity in CA3 was necessary for the antidepressant-like effects of chronic administration of TFF3 in the OB paradigm.

### 2.2. Discussion

In the current study, we investigated the effects of chronic TFF3 treatment on OB-induced depressive-like behaviors in rats. We found that chronic systemic administration of TFF3 (0.1 mg/kg, i.p.) reversed OB-induced depressive-like behaviors and hyperactivity via decreasing the levels of BDNF, pERK1/2, and pCREB in CA3. Finally, we found that inhibition of BDNF or ERK blocked the antidepressant-like effect of TFF3 in the OB paradigm.

Olfactory bulbectomy is a constructive animal model of depression with high predictive validity, because it causes a series of behavioral, physiological, and biochemical changes which are comparable to symptoms of depressed humans and are sensitive to antidepressant treatment [22,23]. Accumulating evidence verified the permanent behavioral deficits in OB rodents [22,23,37]. Hyperactivity in the open field is one parameter of validation for the OB model, which reflects psychomotor agitation, a key symptom in the diagnosis of agitated depression [38]. The augmentation of activity could last at least 20 weeks after surgery [37], which concurs with our results that hyperactivity (present as the number of crossings and rearings in OFT) still existed for three weeks after surgery (see Figure 1C,D, OB-saline group *vs*. sham-saline group). Other behavioral deficits of OB surgery consist of increased immobility in FST and decreased sucrose preference in SPT, which are yet another two important hallmarks of depression [39,40,41]. In this study, we found that OB-induced hyperactivity, increased immobility, and decreased sucrose preference were normalized by chronic administration of TFF3, which is consistent with our previous findings that TFF3 had the antidepressant-like effects tested in the chronic mild stress (CMS) rats model [21]. Furthermore, we found that after treatment for seven days, OB-induced behavioral deficits were normalized by TFF3, whereas venlafaxine only showed a trend but did not produce significant antidepressant-like effect. In addition, we found that chronic administration of TFF3, but not venlafaxine, reduced the immobility in FST in sham rats, which is consistent with our previous finding that acute injection of TFF3 but not venlafaxine decreased the immobility in naive rats [33].

Numerous pieces of evidence indicate that neural networks, including the amygdala, hippocampus, prefrontal cortex, and other regions, participate in the pathophysiology of depression [18,19,42]. In this study, we focused on the hippocampus and investigated the role of BDNF and its downstream targets ERK and CREB, a pathway mainly regulating protein synthesis and synaptic plasticity [43,44], trying to explain the underlying molecular mechanism of the therapeutic ability of TFF3. As a neurotrophic peptide, BDNF is critical for axonal growth, neuronal survival, and synaptic plasticity [45]. Many studies focus on the role of BDNF in neurocircuitry related with depression. Previous data showed that in patients with depression, and animal models, BDNF was decreased in the hippocampus, and antidepressant treatment normalized the expression [27,44,46]. Our findings that OB-induced down-regulation of BDNF in CA3 was significantly blocked by the repeated administration of TFF3 was partially paralleled with previous results. ERK is regulated by Ras-Raf-MEK cascade in response to BDNF activation [27]. Our results showed that OB reduced the pERK1/2 levels in DG. This is in accordance with a previous study that chronic corticosterone, an important causal factor in depression, reduced pERK1/2 expression in DG [26]. We also found that chronic TFF3 renewed the decrease of pERK1/2 induced by OB in hippocampal CA3. Additionally, our results verified the previous findings that stress decreased the phosphorylation of CREB in the CA3 region [47]. These molecular findings confirm the critical role of ERK and CREB in OB-induced depressive-like deficits in the hippocampus. To further determine whether the BDNF-ERK-CREB signaling pathway was necessary for the antidepressant-like effect of TFF3, the rats were co-treated with TFF3 and ANA-12 or U0126 for a week. Our results showed that inhibition of BDNF activity by ANA-12 or pERK1/2 by U0126 in CA3 reversed the therapeutic effects of TFF3 in OB rats. Taken together, these results strongly indicate that the renewal of the BDNF-ERK-CREB signaling in the hippocampal CA3 is, at least in part, the mechanism underlying the antidepressant-like effects of TFF3 in the OB paradigm.

Thus far, although TFF3 has been reported to be involved in several processes in the CNS such as depression [21] and opiate addiction [48], the underlying molecular mechanisms are poorly understood. Our previous study has shown that acute systemic administration of TFF3 with a single dose of 0.1 mg/kg in the basolateral amygdala (BLA) produced an antidepressant-like effect through the phosphatidylinositol 3-kinase (PI3K) /Akt signaling pathway. However, many studies indicated that in OB paradigm, hippocampal BDNF, ERK, and CREB mediated the depressive-like deficits and the efficacy of chronic antidepressants treatment [38,39,49,50]. Studies *in vitro* have shown TFF3 regulated the activation of ERK [24,25]. Thus, in the current study, we focused on the hippocampal BDNF-ERK-CREB pathway. Our results revealed that chronic TFF3 treatment increased the expression of BDNF, pERK1/2, and pCREB in the hippocampal CA3 of OB rats. Several studies have indicated that the change of BDNF levels differs after acute and chronic drug treatment. Chronic, but non-acute, treatment with serotonin reuptake inhibitors increased BDNF levels in the rat brain regions [51,52,53]. In the current OB procedure, TFF3 was chronically injected for seven days but not administrated with a single injection. According to this, we reason that long-term treatment of TFF3 might activate a distinct signaling pathway relative to acute administration. This indicates that TFF3 might produce antidepressant-like effects via multiple signaling pathways in different brain regions.

Nevertheless, it was surprising that no significant effect of venlafaxine on BDNF, pERK1/2, or pCREB was observed. Venlafaxine is a dual serotonin/norepinephrine reuptake inhibitor and exhibits superior efficacy compared with selective serotonin and norepinephrine reuptake inhibitors [54,55]. Several studies have shown that chronic venlafaxine treatment could increase serum or hippocampal BDNF expression in animal models or patients with depression, but such an effect was only detected after long-term administration of venlafaxine for at least three weeks [56,57,58]. Thus, we speculate that in the rat OB model, chronic venlafaxine treatment for more than seven days might be followed by the up-regulation of the BDNF-ERK-CREB signaling pathway, and an antidepressant-like ability. Therefore, the current data showed that TFF3 might be a promising antidepressant candidate with rapid behavioral response.

## 3. Experimental Section

### 3.1. Animals

Male Sprague Dawley rats (240–260 g) were purchased from the Laboratory Animal Center of the Peking University Health Science Center. The rats were housed four per cage before surgery and individually after surgery on a reversal of 12 h/12 h light/dark cycle with controlled temperature (23 ± 2 °C) and humidity (50% ± 5%). Rats had free access to food and water. All the animal procedures were performed according to the National Institutes of Health Guide for the Care and Use of Laboratory Animals and approved by the Biomedical Ethics Committee for animal use and protection of Peking University (LA2010-010).

### 3.2. OB Surgery

After the accommodation phase, the OB procedure was performed as described previously [22,37]. The rats were anesthetized with sodium pentobarbital (50 mg/kg, i.p.) and then fixed in the stereotactic frame. A rostral-caudal midline incision was made on the overlying skin, and two small holes (2 mm diameter) were drilled into the skull 8 mm anterior to the bregma, 2 mm from the midline. Bilateral olfactory bulbs were removed by suction using a blunt needle. A haemostatic sponge was inserted into the cavity to stop bleeding. The incision was then closed with absorbable sutures. Sham rats underwent a similar surgical procedure except for the removal of olfactory bulbs. After surgery, rats were housed individually and injected penicillin (200,000 IU, i.p.) once a day in the first 3 days to prevent infections. All animals were allowed 2 weeks to recover before subsequent processes. During the recovery period, the rats were handled daily to reduce possible stress.

### 3.3. Intracerebral Cannula Implantation

In the last two experiments, rats underwent an intracerebral cannula implantation after OB surgery. The procedure was as described previously [59]. Guide cannulae (23 gauge; Plastics One, Roanoke, VA, USA) were implanted bilaterally 1 mm above hippocampal CA3. The stereotaxic coordinates were the following: anterior/posterior, −3.8 mm; medial/lateral, ±3.8 mm from bregma; dorsal/ventral, −3.2 mm from the skull surface [59,60]. The cannulae were anchored to the skull with stainless-steel screws and dental cement. A stainless-steel stylet blocker was inserted into each cannula to prevent blockage and infection.

### 3.4. Drugs and Treatment

Recombined human TFF3 (rhTFF3) (purity > 98%) was purchased from Beijing Yong Kang Jia Xin Science and Technology Development (Beijing, China). Venlafaxine was purchased from Chengdu Daxi’nan Pharmaceutical Co., Ltd. (Chengdu, China). TFF3 and venlafaxine were freshly dissolved in sterile 0.9% sodium chloride solution. During treatment, saline, TFF3 (0.1 mg/kg, i.p.), and venlafaxine (40 mg/kg, i.p.) were injected at a volume of 1 mL/kg body weight once a day for 7 days consecutively. ANA-12 and U0126 from Sigma (St. Louis, MO, USA) were given to rats 1 h prior to TFF3 each day. ANA-12 was dissolved in 1% dimethylsulfoxide (DMSO) in saline. ANA-12 (0.5 μg per side) or vehicle was microinjected bilaterally into CA3 at a continuous rate of 0.1 μL/min for 5 min. Injector cannulae were removed 2 min after infusions [34,35,61]. U0126 (100 ng per side) was dissolved in 20% DMSO and infused bilaterally into CA3 0.5 μL/min for 1 min. The injectors were kept in place for an additional minute and the stylets were replaced [36].

### 3.5. Behavioral Tests

#### 3.5.1. Sucrose Preference Test

The SPT was conducted according to previous studies [21,62]. Rats were trained to adapt to a 1% sucrose solution (*w*/*v*) for 48 h during the recovery period. Two bottles of 1% sucrose solution were placed in each cage. After adaptation, the rats were deprived of water for 4 h and then submitted to the sucrose preference test, in which rats were housed in individual cages for 1 h and had free access to two bottles containing 1% sucrose solution or water. The bottles were counterbalanced across the left and right. At the end of the 1 h test, sucrose and water consumption (in milliliters) was measured. Sucrose preference (%) was calculated as sucrose consumption/(sucrose consumption + water consumption).

#### 3.5.2. Open Field Test

Locomotor activity in rats was measured using the OFT as described earlier [33]. The open field apparatus consisted of a 75 cm × 75 cm × 40 cm square arena that was divided into twenty-five 15 cm × 15 cm squares on the floor. Each rat was placed in the center of the apparatus and then allowed to explore freely for 5 min, during which the number of rearings and squares crossed was recorded.

#### 3.5.3. Forced Swim Test

The FST was adapted from previous protocols [33,63]. During the adaptive phase, the rats were placed in a 25 cm diameter × 65 cm height plastic cylinder filled to a depth of 45 cm with 23–25 °C water for 15 min. Rats were placed again in the cylinder 24 h later, and the 5 min FST was conducted. Immobility was defined as the minimum movement required to passively keep the animal’s head above the water without other motions.

### 3.6. Tissue Sample Preparation

The procedure was described as previously [33]. After the behavioral tests, rats were decapitated and the tissues from bilateral CA1, CA3 and dentate gyrus (DG) of hippocampus were homogenized (10–15 s × 3, 5 s interval) with an electrical disperser (Wiggenhauser, Sdn Bhd, Berlin, Germany) after being lysed with RIPA lysis buffer (Beyotime Biotechnology, Beijing, China) for 30 min. Subsequently, the homogenate was centrifuged at 10,000× *g* for 20 min and supernatant was collected. All of the above procedures were performed at low temperatures (0–4 °C). Protein concentration was determined using the BCA assay kit (Applygen Technologies, Beijing, China) and then normalized by diluting the samples with RIPA lysis buffer.

### 3.7. Western Blot Assays

Procedures were based on our previous studies [33]. A 5× loading buffer (Applygen Technologies) was added to each sample before being boiled for 5 min. The proteins were loaded to 12% SDS-PAGE and electrophoretically transferred to Immobilon-P transfer membranes (Millipore, Bedford, MA, USA). The blots were blocked for 2 h with blocking buffer (5% BSA in TBST) at room temperature. Membranes were then incubated overnight at 4 °C with the following primary antibodies: BDNF (1:2000; Abcam, Cambridge, UK), pCREB, CREB, pERK1/2, ERK1/2 (1:1000; Cell Signaling, Boston, MA, USA), or β-actin (1:1000; Santa Cruz Biotechnology, Santa Cruz, CA, USA). After washes, the membranes were incubated with horseradish peroxidase-conjugated secondary antibody (goat anti-mouse IgG for β-actin and goat anti-rabbit IgG for the others, 1:5000, Santa Cruz Biotechnology) for 50 min at room temperature. After being washed in TBST four times, the blots were detected by super signal enhanced chemiluminescence substrate (detection reagents 1 and 2, 1:1 ratio, Applygen Technologies) and visualized using Sygene Bio Image system. Band intensities were quantified by Quantity One version 4.4.0 software (Bio-Rad, Hercules, CA, USA). The final results are provided as the ratio of the optical density of specific proteins to the optical density of β-actin.

### 3.8. Statistical Analysis

Data are expressed as mean ± SEM. Statistical analyses were carried out by two-way analysis of variance (ANOVA) followed by Tukey’s *post hoc* test. Values of *p* < 0.05 were considered statistically significant (see Results for details).

## 4. Conclusions

In summary, chronic TFF3 treatment normalized olfactory bulbectomy induced depressive-like behaviors by the regulation of the BDNF-ERK-CREB pathway in the hippocampal CA3, a brain area crucial for depression. The current findings contribute to our understanding of the antidepressant-like effects of TFF3 and the underlying mechanisms in an established animal model of depression. Our findings might be conducive to the development of novel rapid antidepressants in the future.
